# IL-11 in multiple sclerosis

**DOI:** 10.18632/oncotarget.6027

**Published:** 2015-10-07

**Authors:** Xin Zhang, Tracy Putoczki, Silva Markovic-Plese

**Affiliations:** Departments of Neurology and Microbiology and Immunology, University of North Carolina at Chapel Hill, Chapel Hill, North Carolina, USA

**Keywords:** IL-11, multiple sclerosis

The clinically isolated syndrome (CIS) suggestive of multiple sclerosis (MS) is the first clinically evident phase of the disease. An autoimmune response against myelin proteins is considered the key pathogenic process that initiates MS. Peripherally activated myelin-reactive T cells that transmigrate across the blood brain barrier initiate an inflammatory cascade within the CNS that leads to myelin, neuronal and oligodendrocyte loss. Numerous studies have identified that IFN-γ-producing Th1 and IL-17A-producing Th17 cells contribute to the autoimmune response and tissue injury in MS, while IL-4-producing Th2 cells and TGF-β1- and IL-10-producing regulatory T (Treg) cells have an immunomodulatory role.

IL-6 is one of the key cytokines that promotes Th17 differentiation in both mice and humans. IL-11 is an IL-6 cytokine family member that has not been extensively studied in the context of the autoimmune response. In the presence of the ligand-binding receptor subunits IL-6Rα and IL-11Rα, IL-11 and IL-6 bind the same signaling transduction unit gp130, which leads to ternary complexes and activation of similar downstream signaling events. STAT3, a transcription factor involved in Th17 cell differentiation, is predominantly activated in response to IL-6/IL-6R and IL-11/IL-11R axis. While IL-6 is generally accepted as the prototypical pro-inflammatory cytokine, the role of IL-11 in the autoimmune response is poorly understood.

Previous oncology studies have demonstrated that IL-11 is an essential cytokine promoting chronic gastric inflammation and associated gastric, colonic, hepatocellular and breast cancer tumorogenesis through excessive activation of STAT3 [1].

The studies of the inflammatory diseases have demonstrated that intra-articular injection of IL-11 causes joint inflammation, and that mSA/IL-1 acute arthritis was reduced in anti-IL-11 antibody-treated and IL-11Rα1−/− mice [2]. Endogenous IL-11 has proinflammatory effects at sites of IL-13-mediated lymphocytic and eosinophilic tissue inflammation [3]. IL-11 expression was significantly increased in chronic skin lesions in atopic dermatitis [4] and is known to play a critical role in bronchial inflammation [2, 4].

IL-11 is produced by activated astrocytes and IL-11Rα is expressed on oligodendrocytes in MS brain lesions. The chromosomal region containing the IL-11 gene (19q13) is associated with susceptibility to MS [5]. However, IL-11 expression in the inflammatory cells and their roles in the development of the inflammatory response in MS have not been fully elucidated. We recently reported that IL-11 was the most elevated cytokine in the cerebrospinal fluid (CSF) and serum of CIS patients in comparison to control subjects. Moreover, IL-11 serum level are significantly higher during the clinical relapses in comparison to the clinically quiescent phase of RRMS, suggesting the involvement of IL-11 in MS inflammatory responses [6].

**Figure 1:**
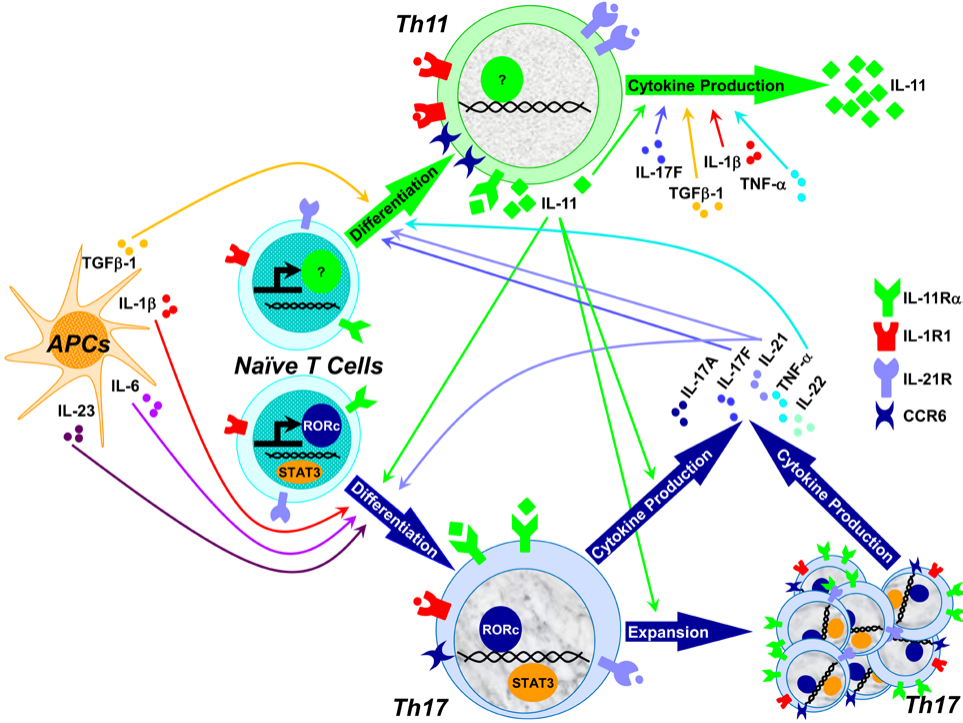
IL-11 selectively induced naïve CD4+ T cell differentiation into Th17 cells, characterized by increased RORc expression and a dose-dependent increase in the percentage of IL-17A- and IL-21-producing CD4+ T cells and the secretion of IL-17A, IL-17F, IL-21 and IL-22. The combination of IL-11 and the established Th17-polarizing cytokines IL-1β, IL6 and IL-23 most effectively induced Th17 cell differentiation. IL-11 induced the expansion of the IL-17A-, IL-21- and IL-22-producing RORc+ Th17 cells. Th17 cytokines IL-17F, IL-21 and TNF-α, as well as TGF-β1 induced the differentiation of IL-11+CD4+ T cells, while IL-17F, TNF-α, TGF-β1, IL-1β, and IL-11 induced IL-11 secretion by memory CD4+ T cells.

IL-11Rα is expressed by multiple cell subsets in peripheral blood mononuclear cells (PBMCs), with predominant expression in T cells [6, 7]. IL-11 selectively induces Th17 cell differentiation in both CIS patients and HCs. The expression of RORc, the percentage of IL-17A and IL-21-producing CD4+ T cells, and the secretion of IL-17A, IL-17F, IL-21 and IL-22 were induced in a dose-dependent manner in naïve CD4+ T cells that were differentiated in the presence of IL-11 [6]. The combination of IL-11 and the established Th17-polarizing cytokines IL-1β, IL6 and IL-23 most effectively induced the Th17 cell differentiation (Figure 1), indicating that IL-11 independently induces Th17 cell differentiation, but also enhances differentiation induced by other Th17-polarizing cytokines [6].

IL-11 also induces the expansion of memory Th17 cells in CIS patients at IL-11 doses detected in the CSF and serum. IL-11 increased the percentage of RORc, IL-17A, IL-21 and IL-22-expressing cells in the CD45RO+ memory CD4+ T cells [6]. In comparison to the naïve CD4+ T cells, memory Th17 cells are more sensitive to low dose of IL-11-mediated expansion, suggesting that IL-11 can amplify the Th17-mediated autoimmune response in the CNS of MS patients (Figure 1) [6].

Early studies have identified stromal cells, including synovicites, lung fibroblasts, epithelial cells, and eosinophils, as the main source of IL-11. Our group has for the first time reported that CD4+ lymphocytes constitute the main cellular source of IL-11 in the peripheral circulation, representing 68.4% of the IL-11-producing PBMCs in CIS patients [6]. IL-11 is also produced by multiple PBMCs, including CD8+ lymphocytes, CD19+ B cells, γδ T cells, and CD56+ NK cells [6]. IL-11+CD4+ T cells expresses higher level of IL-1R1, IL-21R and CCR6, and lower levels of IL-11Rα in comparison to Th1, Th2 and Th17 cells. CCR6 is an essential molecule that mediates the migration of Th17 cells into CNS of mice with experimental autoimmune encephalomyelitis (EAE), an animal model of MS. A significant increase of IL-11 in the CSF from CIS patients may reflect migration of IL-11+CD4+ T cells into the CNS via a CCR6-dependent mechanism.

IL-11 secretion in non-inflammatory cells is induced by IL-17F, IL-22, IL-1β and TGF-β. Recently, we have reported that the differentiation of IL-11+CD4+ T cells was induced by the Th17 cytokines IL-17F, IL-21 and TNF-α, as well as by TGF-β1 (Figure 1) [6]. In addition, IL-17F, TNF-α, TGF-β1, IL-1β, and IL-11 induced IL-11 secretion by memory CD4+ T cells (Figure 1) [6]. The cross-talk between Th17 and IL-11+CD4+ T cells may induce and amplify the autoimmune response in the early stage of MS, and thus represent an attractive therapeutic target in this and other inflammatory diseases.
